# Isopropoxy Benzene Guanidine Kills *Staphylococcus aureus* Without Detectable Resistance

**DOI:** 10.3389/fmicb.2021.633467

**Published:** 2021-02-04

**Authors:** Xiufeng Zhang, Wenguang Xiong, Xianfeng Peng, Yixing Lu, Jie Hao, Zonghua Qin, Zhenling Zeng

**Affiliations:** ^1^National Risk Assessment Laboratory for Antimicrobial Resistance of Animal Original Bacteria, Guangdong Provincial Key Laboratory of Veterinary Pharmaceutics Development and Safety Evaluation, College of Veterinary Medicine, South China Agricultural University, Guangzhou, China; ^2^Guangdong Laboratory for Lingnan Modern Agriculture, Guangzhou, China; ^3^Guangzhou Insighter Biotechnology Co., Ltd., Guangzhou, China

**Keywords:** isopropoxy benzene guanidine, multidrug-resistant *Staphylococcus aureus*, membrane damage, resistance section, transcriptome

## Abstract

Serious infections caused by multidrug-resistant *Staphylococcus aureus* clearly urge the development of new antimicrobial agents. Drug repositioning has emerged as an alternative approach that enables us to rapidly identify effective drugs. We first reported a guanidine compound, isopropoxy benzene guanidine, had potent antibacterial activity against *S. aureus.* Unlike conventional antibiotics, repeated use of isopropoxy benzene guanidine had a lower probability of resistance section. We found that isopropoxy benzene guanidine triggered membrane damage by disrupting the cell membrane potential and cytoplasmic membrane integrity. Furthermore, we demonstrated that isopropoxy benzene guanidine is capable of treating invasive MRSA infections *in vivo* studies. These findings provided strong evidence that isopropoxy benzene guanidine represents a new chemical lead for novel antibacterial agent against multidrug-resistant *S. aureus* infections.

## Introduction

Antibiotic resistance is one of the most prominent public health challenges (Årdal et al., 2019). *Staphylococcus aureus* is the most clinically important multidrug-resistant pathogen and a leading cause of bacteremia, endocarditis, osteomyelitis and skin, and soft tissue infections (Tacconelli et al., 2018; Turner et al., 2019). The alarming increase in the prevalence of global spread clones in *S. aureus* resistant to nearly all antibiotics is a major public health concern (Lakhundi and Zhang, 2018). Hence, there is a dire need to develop novel antimicrobial compounds, as well as are tolerated with low propensity for resistance development (Nambiar et al., 2014).

Nowadays, antimicrobial drug discovery is under constant challenge. The decreasing rate and huge cost of antibiotic discovery has led to alternative strategies being introduced to the clinic (Antibiotics Currently in Global Clinical Development, 2019). Repurposing drugs has emerged as an innovation stream of pharmaceutical development and gained great success in treating various infectious diseases (Austin and Gadhia, 2017; Pushpakom et al., 2019). The majority of recently approved agents against *S. aureus* infections have been developed from existing drug classes, including tetracycline, fluoroquinolone, and pleuromutilin (Talbot et al., 2019). In order to rescue last-resort antibiotics, researchers synthesized vancomycin derivatives by respective or combined modifications, which greatly improved the antibacterial activity or changed the antimicrobial mechanism (Okano et al., 2017; Guan et al., 2018).

As guanidine had strong organic bases and presented hydrophilic in nature, guanidine compounds have been discovered as new promising drugs in both synthetic and medicinal chemistry (Saczewski and Balewski, 2013; Massimba-Dibama et al., 2015). Liu reported that metformin restored tetracyclines susceptibility against multidrug resistant bacteria by promoting intracellular accumulation of doxycycline (Liu Y. et al., 2020). In our previous study, we screened a guanidine compound isopropoxy benzene guanidine (IBG) as anti-*Enterococci* agent by disrupting their cell membrane potential (Zhang et al., 2019). This compound showed low cytotoxicity against human lung epithelial cells and was well tolerated by mice red blood cells. In this study, we characterized the response of *S. aureus* ATCC 29213 to IBG using phenotypic assays and transcriptomics. The probability of IBG on resistance selection was estimated by serial passage assay and whole genome sequencing. The *in vivo* treatment efficacy of IBG was investigated in a mouse septicemia model.

## Materials and Methods

### Bacterial Strains, Growth Conditions

*Staphylococcus aureus* strain ATCC 29213, methicillin-resistant *S. aureus* (MRSA) ATCC 43300, 105 clinical *S. aureus* isolates (including 55 MRSA strains), and 8 Gram-negative strains were used in this study. *Acinetobacter baumannii* and *Klebsiella pneumonia* were grown in Cation-Adjusted Mueller-Hinton (MH) broth, all other strains were grown in MH broth. Multilocus sequence typing (MLST) was conducted according to the reference MLST database.

### Antimicrobial Agents and Chemicals

Vancomycin (VAN), gentamicin, ciprofloxacin (CIP), linezolid, and amikacin were purchased from Sangon Biotech (Shanghai, China). Isopropoxy benzene guanidine (IBG) (batch number 20150506, content 99.9%) was synthesized by Guangzhou Insighter Biotechnology (Guangzhou, China). Dimethyl Sulphoxide (DMSO) (Dmreagent, Tianjing, China) was utilized as solvent to dissolve IBG. Anti-infective detergent benzalkonium chloride (BAC) was purchased from Aladdin Industrial Corporation (Shanghai, China). SYTOX^®^ green nucleic acid stain agents and 3,3′-diethyloxacarbocyanine iodide [DiOC_2_(3)] (Thermo Fisher Scientific, Germany) were used as molecular probes.

### Antibacterial Test

The MIC and MBC of IBG were determined by broth microdilution according to CLSI guidelines (Clinical and Laboratory Standards Institute, 2018). The MBC was defined as the lowest concentration where a 99.9% colony count reduction was observed (Mohammad et al., 2015). The presence of fetal bovine serum (Tianhang Biotechnology, Zhejiang, China) on IBG activity against *S. aureus* was also tested. Experiments were performed in triplicates.

### Antibiotic Synergy Test

The checkerboard method was used for determining synergy of IBG with conventional antibiotics (Falagas et al., 2019). The interaction between two compounds was defined as synergy if FICI < 0.5, addition if 0.5 ≤ FICI ≤ 1, no interaction if 1 < FICI ≤ 4, antagonism if FICI > 4.

### Killing Kinetics Assay

The time-dependent killing for *S. aureus* ATCC 29213 and MRSA 43300 with IBG, VAN at 10 × MIC was investigated previously (AbdelKhalek et al., 2016). The OD_600nm_ was measured to determine bacterial lysis when *S. aureus* (OD_600nm_ ∼0.4) treated with 10 × MIC of IBG, VAN, and BAC (positive control) for 4 h (Kim et al., 2018). All the experiments were replicated.

### Resistance Studies

In order to select IBG-resistant mutants, ∼10^10^ CFU of *S. aureus* ATCC 29213 cells were plated onto MH agar containing 2.5×, 5×, and 10×MIC of IBG. After 48 h of incubation at 37°C, resistant colonies were calculated and the MICs of IBG were determined. When this approach proved unsuccessful, development of resistant mutants by serial passage in liquid medium was conducted previously (Ling et al., 2015). The bacteria culture (OD_600_ = 0.01) was treated with IBG or CIP at different concentrations. Cells were incubated at 37°C and passaged at 24 h intervals in the presence of IBG or CIP. The MIC was determined by broth microdilution. Experiments were performed with three replicates (SP1, SP2, and SP3).

The genomic DNA from the strains with elevated MIC of IBG was extracted using a Hipure bacterial DNA kit (Magen, Shanghai, China). A paired-end sequencing library (2 bp × 250 bp) was created using a VAHTS Universal DNA Library Prep kit for Illumina^®^ (Illumina, San Diego, CA, United States) and sequenced on an Illumina HiSeq system (Illumina Inc.). Processed reads were *de novo* assembled into draft genomes using CLC Genomics Workbench 10.1 (CLC Bio, Aarhus, Denmark) and annotated by using Prokka pipeline. These genomes were subjected to SNP analysis by utilizing snippy pipeline. *S. aureus* ATCC 29213 genome of the starting strain was used as a reference genome in SNP analysis.

### Membrane Potential Assay

To examine the perturbation of the cell membrane of *S. aureus* by IBG, the membrane potential of the cells was measured by fluorescence spectrometry using fluorescent probe DiOC_2_(3), as described previously (Wang et al., 2017). Bacterial cells were energized by the addition of glucose to establish a proton motive force (negative and basic inside the cell). This led to an increase in fluorescence associated with aggregation of the DiOC_2_(3). Upon addition of the ionophore CCCP, the Δψ was dissipated and the fluorescence intensity dropped to the level before addition of glucose. All assays were performed at least twice.

### Membrane Permeability Assay

To confirm the integrity of the bacterial membranes, we performed an assay based on the uptake of the fluorescent dye with SYTOX Green (Kim et al., 2018). The fluorescence was measured using a multifunctional microplate reader, with excitation and emission wavelengths of 485 and 525 nm, respectively. All experiments were conducted in duplicate.

### Transmission Electron Microscopy

Morphological appearance of the cell membrane of *S. aureus* (ATCC 29213) treated with 10 × MIC IBG was observed using a JEOL 1200EX transmission electron microscopy (TEM) (Li et al., 2016).

### Transcriptome Analysis

*Staphylococcus aureus* ATCC 29213 cells (OD_600_∼0.4) were treated with 10 × MIC IBG for 4 h. Then bacteria was collected and preserved with RNA protect (Qiagen, United States). Total RNA of each sample was extracted using TRIzol Reagent (Invitrogen)/RNeasy Mini Kit (Qiagen). Control samples were collected from an antibiotic-free culture. RNA sequencing was conducted by High-Throughput Sequencing Facility at the GENWIZ lnc (Jiangsu, China). Raw sequence data were underwent quality control using Cutadapt (V1.9.1) and FastQC (V0.10.1). Clean data were aligned with the reference genome of *S. aureus* NCTC 8325 (NCBI accession number NC_007795.1) *via* software Bowtie2 (v2.1.0) and the gene expression level were estimated by HTSeq (v0.6.1p1). The calculation of fragment per kilobase of exon per million fragments mapped (FPKM) for all genes were performed through Cufflinks (v2.2.1) software. Differential expression genes (DEGs) were screened out by using the DESeq Bioconductor package and defined as those with a change in expression of >twofold and a corresponding false discovery rate (FDR) of <0.05. The gene ontology terms and functional pathways were annotated *via* Gene Ontology (GO) and Kyoto Encyclopedia of Genes and Genome (KEGG), respectively. Cell-PLoc 2.0 was used to analyze the subcellular localization of DEGs (Chou and Shen, 2010).

### Mouse Sepsis Protection Model

Animal studies were carried out at animal laboratory center of South China Agricultural University and approved by the Animal Research Committee of South China Agricultural University (2018030). All animal studies were performed with specific-pathogen-free female KM mice (Southern Medical University, Guangdong, China), 6–8-weeks old, weighing 20 ± 2 g.

A mouse septicemia protection assay was used to assess *in vivo* treatment efficacy of IBG (Thangamani et al., 2016). KM female mice were treated intraperitoneally with a dose of 40 mg/kg IBG. After 24 h, KM female mice were infected with 0.1 ml of bacterial suspension (MRSA YXMC004P) *via* tail vein injection, a concentration that achieves about 90% mortality within 18 days after infection. At 0.5 h and 24 h post infection, mice (18 per group) were treated with 40 mg/kg IBG, 15 mg/kg VAN and PBS via intraperitoneal injection. Mice were monitored for 18 days after MRSA infection and the statistical analysis was performed by non-parametric log-rank test.

### Statistical Analyses

Statistical analysis was performed using GraphPad Prism 5 and SPSS software. All data were presented as the mean ± s.d.

### Data Availability

The whole-genome resequencing for eight *S. aureus* strains involved in resistance studies have been deposited in GenBank under accession numbers: VUKQ00000000, VUKK00000000, VUKL00000000, VUKM00000000, VUKO00000000, VUKP00000000, VUKR00000000, and VUKS00000000. RNA-seq data have been deposited in the NCBI’s Sequence Read Archive with accession number PRJNA557004.

## Results

### Antimicrobial Activity of IBG

**FIGURE 1:**
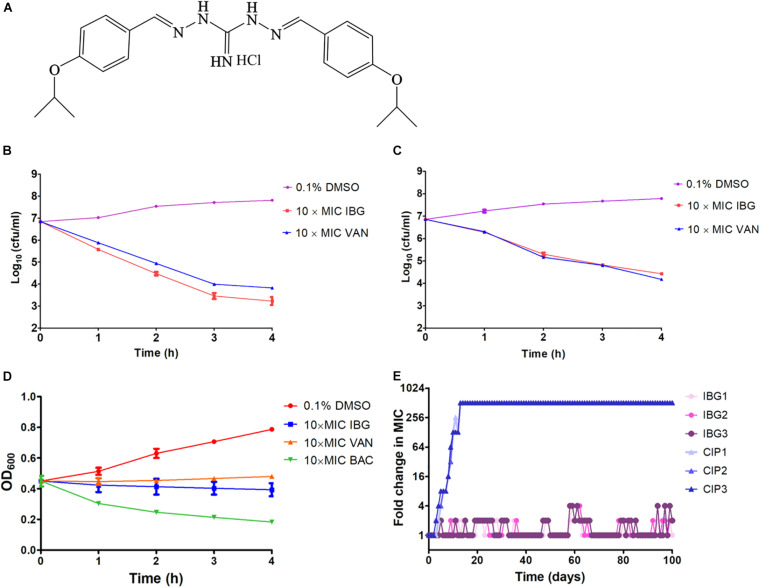
IBG inhibits *S. aureus* growth without detectable mutant development. **(A)** Structure of IBG. **(B)** Viability of *S. aureus* ATCC 29213 treated with IBG or VAN. **(C)** Viability of MRSA ATCC 43300 treated with IBG or VAN for 4 h. **(D)** IBG treatment could not result in *S. aureus* ATCC 29213 lysis; **(E)** Appearance of spontaneous IBG and CIP-resistant *S. aureus* mutants over 100 days of serial passages. IBG, isopropoxy benzene guanidine; VAN, vancomycin; CIP, ciprofloxacin; Individual data points (*n* = 3 biologically independent experiments) and mean ± SD. are shown.

The structure of IBG was shown in Figure 1A. IBG exhibited potent activity against all tested *S. aureus* with the MIC range of 0.125–4 μg/ml (Table 1). However, IBG was inactive against Gram-negative bacteria (Supplementary Table 1). Besides, in the presence of 10% FBS, the MIC range increased eightfold. The MBC range for IBG against *S. aureus* strains harboring different MLST types was 4–8-fold of their MICs (Table 2). The kill kinetics of IBG was similar to VAN, which greatly reduced the number of bacteria within 4 h (Figures 1B,C). However, IBG and VAN did cause *S. aureus* lysis (Figure 1D). In addition, IBG exhibited addition activity (0.5 < FICI < 1) with gentamicin or amikacin when tested against *S. aureus* ATCC 29213, MRSA ATCC 43300 (Supplementary Figure 1).

**TABLE 1:** Activity of IBG against *Staphylococcus aureus*.

Organism	IBG MIC (μg/ml)	VAN MIC (μg/ml)
*S. aureus* ATCC 29213 (MSSA)	4	1
*S. Aureus* 29213 + 10% FBS	32	1
MRSA ATCC 43300	4	1
MRSA 43300 + 10% FBS	32	1
Clinical MSSA (50)	0.125–4	0.5–1
Clinical MRSA (55)	2–4	0.5–1

*IBG, isopropoxy benzene guanidine; VAN, vancomycin; FBS, fetal bovine serum; -, undetected. MIC, Minimum inhibitory concentration; MSSA, methicillin-sensitive *S. aureus*; MRSA, methicillin-resistant *S. aureus*.*

**TABLE 2:** MBC of IBG against *Staphylococcus aureus*.

Organism	MLST	Spa type	MIC (μg/ml)	MBC (μg/ml)
MSSA ATCC 29213			4	16
MRSA ATCC 43300			4	16
MSSA GDM4P080P	9	899	4	32
MSSA GDE5P004P	9	1775	2	16
MSSA 131372	22	–	4	16
MSSA 131373	7	–	4	16
MSSA 131374	188	–	4	16
MSSA 131390	59	–	4	16
MSSA 131391	5	–	4	16
MSSA 131403	944	–	4	16
MSSA 131440	15	–	4	16
MSSA 131447	6	–	0.125	1
MSSA 131448	88	–	4	16
MSSA 131449	188	–	4	16
MRSA THPS19-2	9	899	4	32
MRSA GDT4P196P	9	1939	4	16
MRSAGDC6P096P	398	34	4	16
MRSA SHP6P028P	398	571	2	8
MRSA 131371	338	–	4	16
MRSA 131404	45	–	4	16
MRSA 131405	59	–	4	16
MRSA 131436	1	–	4	16

*MBC, Minimum bactericidal concentration; IBG, isopropoxy benzene guanidine; –, undetected; MIC, Minimum inhibitory concentration; MSSA, methicillin-sensitive *S. aureus*; MRSA, methicillin-resistant *S. aureus*.*

### IBG Had a Low Probability of Resistance Selection in *S. aureus*

We were unable to obtain IBG-resistant mutants by plating 10^10^ CFU of *S. aureus* ATCC 29213 on agar containing 2.5×, 5× or 10× MIC of IBG. Similarly, serial passage of three independent *S. aureus* 29,213 cultures for 100 days treated with IBG yielded only putative mutants with two or fourfold greater resistance to IBG, whereas serial passage in CIP for 100 days generated strains that were 512-fold more resistant (Figure 1E and Table 3). A total of 24 mutation genes were identified and most mutations genes encoded products related to catalytic activity, binding and proton transmembrane transporter activity (Supplementary Table 2). In addition, *atpE*, *pcrA*, and *walR* genes were responsible for the reduced susceptibility of IBG.

**TABLE 3:** Antimicrobial susceptibility of *S. aureus* ATCC 29213 mutants isolated by serial passage for 100 days.

Mutant strain	Mutated gene	MIC (μg/ml)				
	
		IBG	VAN	LNZ	CIP	GEN
SP1-1		4	1	1	0.25	0.5
SP1-58	*pnp_1*, *atpE*, *walR*, *pcrA*, *ybiV*, *Org1_01939*, *Org1_01695*, *Org1_01489*	8	2	0.5	0.25	0.5
SP1-99	*rsmG*, *atpE*, *dtd3*,*walR*, *pcrA*, *Org1_01695*, *Org1_01489*, *prkC*, *lip2*, *mnhG1*	8	2	0.5	0.5	0.5
SP2-1		4	1	1	0.25	0.5
SP2-58	*rpoE*, *atpE*, *walR*, *pcrA*, *ybiV*, *Org1_01695*, *Org1_01701*, *Org1_01489*, *Org1_01939*, *Org1_00143*, *pnp_1*	16	2	0.5	0.5	0.5
SP2-100	*nusG*, *atpE*, *pcrA*, *isdG_2*, *Org1_00312*	8	2	1	0.25	0.5
SP3-1		4	1	1	0.25	0.5
SP3-34	*atpE*, *walR*, *pcrA*, *ybiV*, *Org1_01489*, *lip2*	8	2	1	0.5	0.5
SP3-66	*Org1_00874*, *atpE*, *Org1_00657*, *walR*, *pcrA*, *ybiV*, *Org1_01695*, *Org1_01489*, *Org1_01939*, *Org1_00143, pnp_1*	8	2	0.5	0.25	0.5
SP3-100	*Org1_00874*, *atpE*, *Org1_00657*, *walR*, *pcrA*, *ybiV*, *sasA*, *Org1_01939*, *Org1_00143, tcaR*, *pnp_1*, *glpK*	8	2	0.5	0.25	0.5

*MIC, minimum inhibitory concentration; IBG, isopropoxy benzene guanidine; VAN, vancomycin; LNZ, linezolid; CIP, ciprofloxacin; GEN, gentamicin. SP1, SP2, and SP3 indicate three independent *S. aureus* 29213 cultures. Kb, *S. aureus* 29213 cells treated with 0.1% DMSO.*

### IBG Disrupted Cell Membrane in Multiple Ways

**FIGURE 2:**
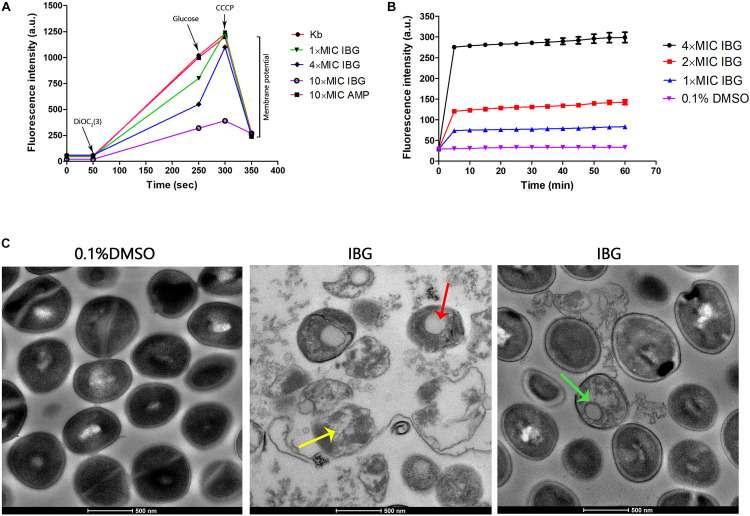
IBG exerts its antibacterial action on the cell membrane of *S. aureus*. **(A)** IBG dissipates the membrane potential of *S. aureus* ATCC 29213. *P* values were determined using a non-parametric one-way ANOVA. *P* values of (*≤0.05) (**P ≤ 0.01) are considered as significant. **(B)** Uptake of SYTOX Green by exponential-phase *S. aureus* ATCC 29213 cells treated with IBG. **(C)** Transmission electron micrographs of *S. aureus* cells in response to IBG. Scale bars, 500 nm. These arrows indicate damage to the cell membrane by IBG. Kb, *S. aureus* ATCC 29213 control cell treated with 0.1% DMSO.

A large reduction in the magnitude of the generated membrane potential was observed in the IBG-treated group, compared to that of the untreated cells and cells in the presence of ampicillin (Figure 2A). Therefore, IBG involved disruption of the inner membrane of bacteria. The membrane disruption properties of IBG were monitored by the SYTOX Green uptake. The fluorescence value clearly increased treated with IBG, even at a concentration of 1× MIC, which indicated IBG could cause the damage of cell plasma membrane (Figure 2B). TEM results showed that IBG treatment caused remarkable morphological changes in *S. aureus* cells, such as mesosome-like structures, cells with ruptured wall and cytoplasmic membrane were found. Even worse, the cytoplasmic contents of some cells were released to the extracellular medium (Figure 2C).

### IBG Regulated the Expression of Membrane Related Genes

**FIGURE 3:**
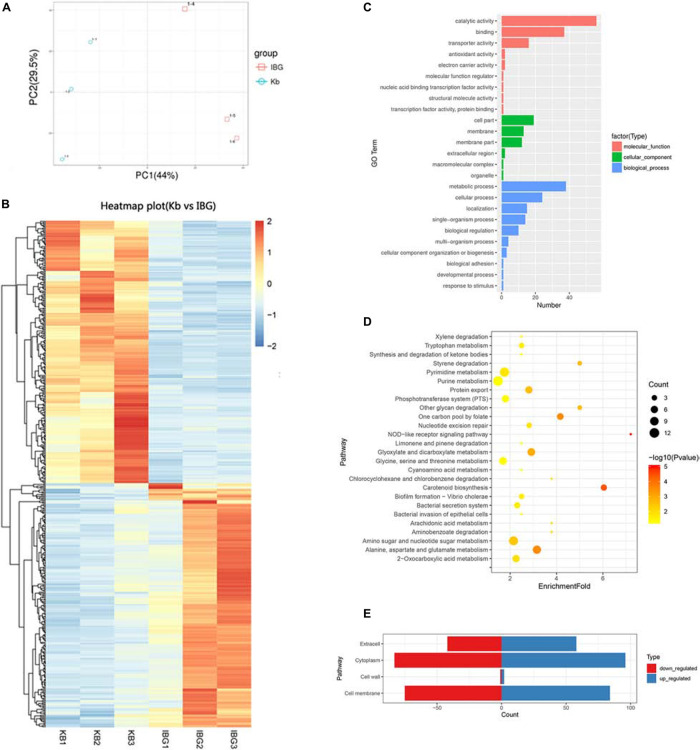
Transcriptome analysis of DEGs in response to IBG stress. **(A)** PCA results. **(B)** Heat map representation of DEGs; **(C)** Go enrichment analysis of DEGs. **(D)** KEGG enrichment analysis of DEGs. **(E)** Subcellular localization of DEGs. DEGs, different expression genes; While all selected genes in RNA-seq data had FDR value of <0.05, which meant significant difference.

**FIGURE 4:**
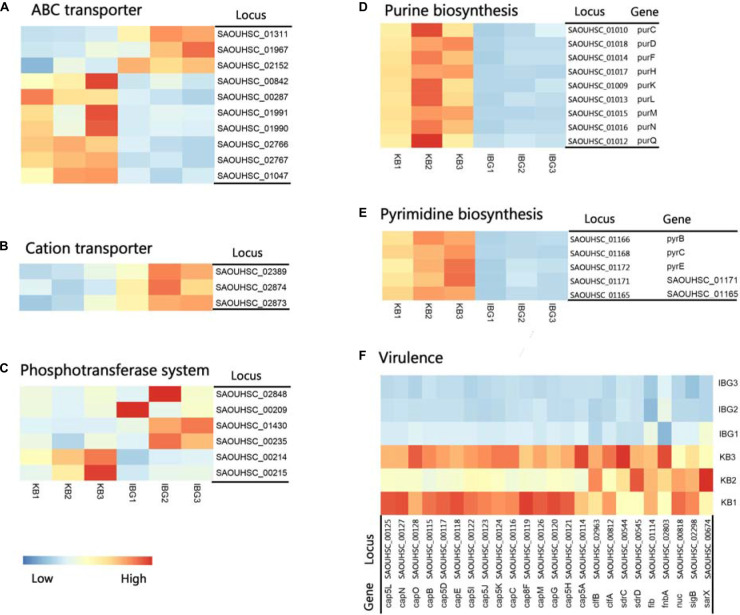
Heat map representation of DEGs involved in main pathways. **(A)** DEGs related to ABC transporters. **(B)** DEGs involved in cation transporters; **(C)** DEGs related to phosphotransferase system. **(D)** DEGs involved in purine biosynthesis pathway. **(E)** DEGs involved in pyrimidine biosynthesis pathway. **(F)** DEGs involved in virulence. All genes represented have a >twofold change in expression treated with IBG relative to their expression in control samples.

The principal component analysis (PCA) analysis revealed that IBG successfully separated treated samples from untreated samples (Figure 3A). Compared to the control group, IBG treatment led to an up-regulation of 230 and down-regulation of 214 DEGs (Figure 3B). According to the GO and KEGG enrichment analysis, IBG treatment caused the changed expression of genes involved in basic metabolic processes, including purine metabolism, pyrimidine metabolism, amino sugar and nucleotide sugar metabolism, alanine, aspartate and glutamate metabolism (Figures 3C,D). Notably, more than a third of DEGs (159/444) located on cell membrane, which mostly related to membrane transport function (Figure 3E). These genes were associated with osmotolerance, including those encoding efflux pumps, enzymes involved in ABC transporters, cation transporters and phosphotransferase system (PTS) (Figures 4A–C). Specifically, virulence, purine and pyrimidine biosynthesis pathway related genes in *S. aureus* were down-regulated under IBG treatment (Figures 4D–F).

### IBG Rescued Mice From MRSA Septicemic Infection

Mice were infected with MRSA at a dose (3.0 × 10^6^ CFU/mouse) that led to a 83.3% mortality (Supplementary Figure 2). The survival rate of infected mice was 66.6 and 83.3% treated with IBG or VAN, respectively. These results suggested that IBG was efficacious in protecting mice from septicemic MRSA infection.

## Discussion

*Staphylococcus aureus* infections pose a significant challenge to public health due to the diminishing arsenal of effective antibiotics available. The development of novel antibacterial discovery has not kept pace with the rapid emergence of bacterial resistance to numerous antibiotics. Some researchers have proved that repurposing drug was the less time-consuming and more financially way to identify new antibiotics (Austin and Gadhia, 2017). In this study, we found that a guanidine compound displayed potent bactericidal activity against *S. aureus* by targeting cell membrane.

We demonstrated that IBG possessed antibacterial activity against multi-resistant *S. aureus*, including MRSA. Compared to the robenidine analog 16 (3-OCH_3_) and 26 [4-CH(CH_3_)_2_] reported previously (Abraham et al., 2016), IBG only had the isopropoxy group [3-OCH(CH_3_)_2_] replacement in benzene ring, but showed the most active against MRSA. This finding was consistent with the antimicrobial activity of IBG against *Enterococci* (Zhang et al., 2019). Additionally, IBG maintained their activity against *S. aureus* isolates exhibiting resistance to different class of antibiotics, which indicated cross-resistance between IBG and these antibiotics is unlikely to occur.

Antibiotic resistance can evolve through sequential accumulation of multiple mutations. Hence, we performed laboratory evolution experiments to assess the propensity of bacteria to develop drug resistance (Toprak et al., 2011). We found that MIC of IBG was almost unchanged over 100 days, whereas the MIC of CIP quickly increased, which is consistent with the report that quinolones can easily induce bacteria to develop drug resistance (Ling et al., 2015). These results demonstrated that *S. aureus* cannot easily develop resistance to IBG. To identify the genetic changes responsible for IBG resistance, we performed whole-genome sequence of seven hyposensitive *S. aureus* strains to IBG. All strains had two mutation genes (*aptE* and *pcrA*) and six of them had a mutation in *walR* gene. Gene *atpE* encodes subunit C of the ATP synthase which utilizes energy stored in the transmembrane electrochemical gradient to synthesize ATP and has been reported to involve in antibiotic resistance (Lamontagne Boulet et al., 2018). PcrA is an ATP-driven 3′–5′ DNA helicase which is involved in DNA repair and plasmid rolling circle replication (Dillingham et al., 2001; Mhashal et al., 2016). Mutations in *walR* locus in *S. aureus* have been shown to relate to reduced susceptibility of vancomycin, which was also found in our study. WalR is a member of the two-component regulatory system WalKR that regulates genes involved in antibiotic resistance, autolysis, biofilm formation and cell wall metabolism (Howden et al., 2011). In addition, there is a *rpoE* mutation in the day 34 of SP2. RpoE (δ factor) is the DNA-dependent RNA polymerase (RNAP) subunit and works as a part of transcription machinery in *S. aureus*. It has been reported that *rpoE* can be involved in orchestrating the ability of *S. aureus* to react and adapt to environmental changes and play a critical role in virulence (Weiss et al., 2014).

We previously confirmed that IBG displayed potent bactericidal activity against *Enterococcus* by disrupting the cell membrane potential^12^. This was also observed in *S. aureus.* The results showed IBG could disrupt the bacterial cell membranes. Similar findings were reported in a previous study in which MRSA strains were treated with robenidine analog NCL195 or techniques (Muthaiyan et al., 2012; Ogunniyi et al., 2017). Moreover, the micrograph indicated that the ultra-structural changes caused by IBG treatment might be irreversible. Perturbation of its structure by IBG maybe the crucial reason for its lethal action on *S. aureus*. However, death is not accompanied by a decline in culture absorbance.

Analysis of transcriptomic changes caused by IBG showed that the expression of genes associated with ABC transport and PTS transporter were remarkable changed. It was found that these transporter systems were associated with nutrient uptake and the export of toxins and antibiotics (Fleury et al., 2009; Liu M. et al., 2020). This may hint the disruption of the membrane potential could hinder the establishment and maintenance of essential energy sources for cell functioning. In addition, IBG treatment also repressed virulence and nucleotide biosynthesis. The down-regulation of virulence factor genes including *sigB*, capsules polysaccharides, might be very likely as a secondary effect for bacteria to survive (Shinji et al., 2011; Tuchscherr et al., 2015; Wang et al., 2019). Purine and pyrimidine biosynthesis pathway genes, crucial for cell growth *via* DNA and RNA synthesis, were down-regulated in IBG treatment group (Li et al., 2018). This was probably induced by the derived effects of increased membrane permeability. Its membrane-targeting actions toward bacteria might contribute to the lack of drug resistance during prolonged laboratory culture in its presence.

The effect with IBG was decreased at 10% FBS, which suggests a high level of protein binding of IBG. This was also observed in robenidine analogs (Abraham et al., 2016). But it does not necessarily render these compounds ineffective *in vivo*. Our results showed IBG could used as a therapeutic agent against mice MRSA systemic infection. Furthermore, IBG demonstrated additive activity when combined with antibiotics traditionally used to treat systemic MRSA infections. This is important given the emergence of resistance to systemic antimicrobials currently used in the clinic; pairing these antibiotics with IBG may improve the morbidity associated with bacterial infections and stymie the rate at which resistance to these antibiotics arises.

## Conclusion

In the summary, we first identified that IBG possesses potent antimicrobial activity against clinical isolates of *S. aureus* by targeting cell membrane. IBG had a low probability of resistance selection and considerable efficacy. IBG has the promising potential to become a new class of antimicrobials for the treatment of Gram-positive bacterial infections.

## Data Availability Statement

The datasets presented in this study can be found in online repositories. The names of the repository/repositories and accession number(s) can be found in the article/Supplementary Material.

## Ethics Statement

The animal study was reviewed and approved by the Animal Research Committee of South China Agricultural University (2018030). Animal studies were carried out at the animal laboratory center of South China Agricultural University.

## Author Contributions

ZZ conceived and designed the project. XZ and WX drafted the manuscript. XP and ZQ worked on the synthesis of compound IBG. YL and JH preformed the antimicrobial susceptibility tests, time-kill assay, and checkerboard assay. XZ carried out the RAN-Seq and data analysis. All authors read and approved the final manuscript.

## Conflict of Interest

XP and ZQ was employed by company Guangzhou Insighter Biotechnology Co., Ltd. The remaining authors declare that the research was conducted in the absence of any commercial or financial relationships that could be construed as a potential conflict of interest.
