# Supplementary material for: Isopropoxy Benzene Guanidine Kills Staphylococcus aureus Without Detectable Resistance
Source: Front Microbiol. 2021 Feb 4;12:633467. doi: 10.3389/fmicb.2021.633467 (PMC7890237; doi:10.3389/fmicb.2021.633467)
Supplement: Supplementary file 1 [file Data_Sheet_1.pdf]

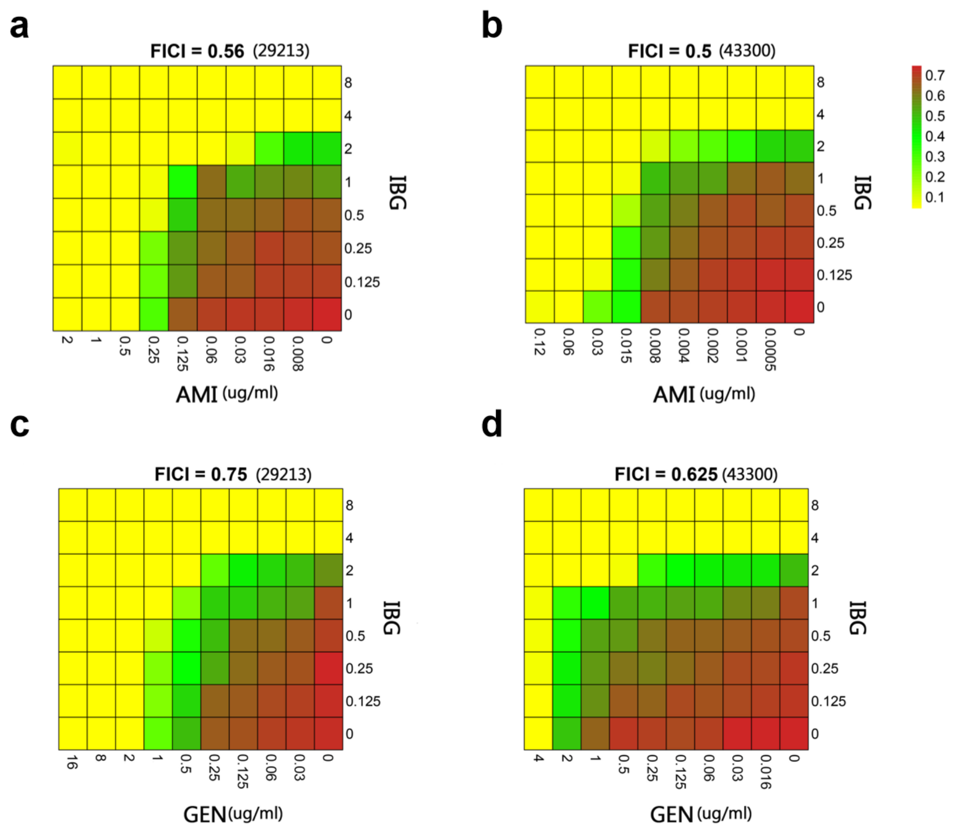

Fig S1 Potentiation of IBG partnered with amikacin, gentamicin against *S. aureus*.

a, The microdilution chequerboard assays of IBG and amikacin against *S. aureus* ATCC 29213. b, The microdilution chequerboard assays of IBG and amikacin against MRSA ATCC 43300. c, The microdilution chequerboard assays of IBG and gentamicin against *S. aureus* ATCC 29213. d, The microdilution chequerboard assays of IBG and gentamicin against MRSA ATCC 43300.

IBG, isopropoxy benzene guanidine; VAN, vancomycin; AMI, amikacin; GEN, gentamicin; The colour gradient represents the bacterial cell density estimated by OD_{600} of biological replicates.

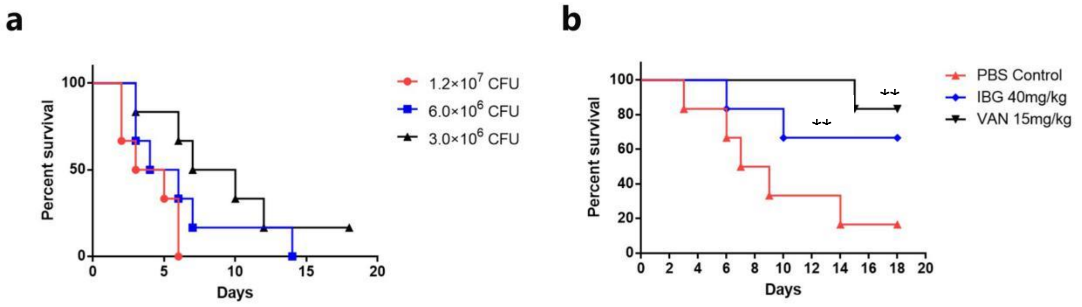

Fig S2 IBG is effective in a mouse model of MRSA septicemic infection

a, Different MRSA infection doses in mouse septicemic model b, Survival curves showing efficacy in a mouse septicemic infection model when treated with different drugs. A log rank test was performed using 95% confidence intervals and the statistical significance was calculated in order to compare treated to control groups. P values of (* ≤ 0.05) (**P ≤ 0.01) are considered as significant.

Supplementary Table S1: Activity of IBG against Gram-negative bacteria

Organism and genotype	IBG MIC (μg/ml)
Escherichia coli ATCC25922	>256
Salmonella ATCC14028	>256
Klebsiella pneumonia ATCC 4752	>256
Acinetobacter baumannii ATCC 19606	>256
Pasteurella multocida CVCC399	>256
Haemophilus parasuis CVCC13R	>256
Serratia marcescens CVCC52721	>256
Clinical *Pseudomonas aeruginosa*	>256

Supplementary Table 2 Amino acid changes located in open reading frames after *in vitro* selection with IBG

Strains	Day of passage	Mutation position	Mutation type	Mutated gene	Function	Base change	Amino acid change
SP1	58	127787	missense variant	*pnp_1*	polyribonucleotide nucleotidyltransferase	247C>G	Arg83Gly
SP1	58	28077	missense variant	*atpE*	ATP synthase subunit c	62A>T	Asn21Ile
SP1	58	31303	missense variant	*walR*	Transcriptional regulatory protein walR	319C>T	Arg107Cys
SP1	58	33408	missense variant	*pcrA*	ATP-dependent DNA helicase PcrA	761A>G	Asp254Gly
SP1	58	34705	missense variant	*ybiV*	sugar phosphatase YbiV	527C>T	Ser176Leu
SP1	58	43553	missense variant	*Org1_01695*	hypothetical protein	95C>A	Pro32Gln
SP1	58	61600	missense variant	*Org1_01489*	hypothetical protein	206G>C	Gly69Ala
SP1	58	76543	missense variant	*Org1_01939*	putative universal stress protein	247G>A	Val83Ile
SP1	58	85845	missense variant	*Org1_00143*	hypothetical protein	42G>A	Met14Ile
SP1	99	1594	stop gained	*rsmG*	ribosomal RNA small subunit methyltransferase G	153T>G	Tyr51*

Strains	Day of passage	Mutation position	Mutation type	Mutated gene	Function	Base change	Amino acid change
SP1	99	28077	missense variant	*atpE*	ATP synthase subunit c	62A>T	Asn21Ile
SP1	99	28879	missense variant	*dtd3*	D-aminoacyl-tRNA deacylase	469G>A	Gly157Ser
SP1	99	31189	missense variant	*walR*	transcriptional regulatory protein walR	205C>T	Arg69Cys
SP1	99	33408	missense variant	*pcrA*	ATP-dependent DNA helicase PcrA	761A>G	Asp254Gly
SP1	99	43795	stop gained	*Org1_01695*	hypothetical protein	665G>A	Trp222*
SP1	99	61414	frameshift variant	*Org1_01489*	hypothetical protein	22_41delTTTCGAAAAGAAATTCTACA	Phe8fs
SP1	99	65156	missense variant	*prkC*	serine/threonine-protein kinase PrkC	1610A>T	Lys537Ile
SP1	99	82809	missense variant	*lip2*	lipase 2	1219G>A	Val407Ile
SP1	99	128200	missense variant	*mnhG1*	Na(+)/H(+) antiporter subunit G1	74C>A	Ala25Asp

Strains	Day of passage	Mutation position	Mutation type	Mutated gene	Function	Base change	Amino acid change
SP2	58	8863	missense variant	*rpoE*	putative DNA-directed RNA polymerase subunit delta	189C>A	Asn63Lys
SP2	58	28077	missense variant	*atpE*	ATP synthase subunit c	62A>T	Asn21Ile
SP2	58	31303	missense variant	walR	transcriptional regulatory protein walR	319C>T	Arg107Cys
SP2	58	33408	missense variant	*pcrA*	ATP-dependent DNA helicase PcrA	761A>G	Asp254Gly
SP2	58	34705	missense variant	*ybiV*	Sugar phosphatase YbiV	527C>T	Ser176Leu
SP1	58	43553	missense variant	*Org1_01695*	hypothetical protein	95C>A	Pro32Gln
SP1	58	51219	missense variant	*Org1_01701*	hypothetical protein	29G>C	Gly10Ala
SP1	58	61600	missense variant	*Org1_01489*	hypothetical protein	206G>C	Gly69Ala
SP2	58	76543	missense variant	*Org1_01939*	putative universal stress protein	247G>A	Val83Ile
SP2	58	84845	missense varian	*Org1_00143*	hypothetical protein	42G>A	Met14Ile
SP2	58	127787	missense variant	*pnp_1*	polyribonucleotide nucleotidyltransferase	247C>G	Arg83Gly

Strains	Day of passage	Mutation position	Mutation type	Mutated gene	Function	Base change	Amino acid change
SP2	100	20011	frameshift variant	*nusG*	transcription termination/ antitermination protein NusG	193delA	Thr65fs
SP2	100	28077	missense variant	*atpE*	ATP synthase subunit c	62A>T	Asn21Ile
SP2	100	33408	missense variant	*pcrA*	ATP-dependent DNA helicase PcrA	761A>G	Asp254Gly
SP2	100	33662	missense variant	*isdG_2*	heme oxygenase (staphylobilin-producing)	409T>C	Tyr137His
SP2	100	34665	frameshift variant	*Org1_00312*	hypothetical protein	1150dupT	Ser384fs
SP2	100	433559	missense variant	*Org1_01695*	hypothetical protein	901G>T	Val301Leu
SP3	34	1594	stop gained	*rsmG*	ribosomal RNA small subunit methyltransferase G	153T>G	Tyr51*
SP3	34	28077	missense variant	*atpE*	ATP synthase subunit c	62A>T	Asn21Ile
SP3	34	28879	missense variant	*dtd3*	D-aminoacyl-tRNA deacylase	469G>A	Gly157Ser

Strains	Day of passage	Mutation position	Mutation type	Mutated gene	Function	Base change	Amino acid change
SP3	34	31189	missense variant	*walR*	Transcriptional regulatory protein walR	205C>T	Arg69Cys
SP3	34	33408	missense variant	*pcrA*	ATP-dependent DNA helicase PcrA	761A>G	Asp254Gly
SP3	34	61489	missense variant	*Org1_01489*	hypothetical protein	95C>A	Pro32Gln
SP3	34	82809	missense variant	*lip2*	lipase 2	1219G>A	Val407Ile
SP3	66	10208	missense variant	*Org1_00874*	hypothetical protein	82G>T	Ala28Ser
SP3	66	28077	missense variant	*atpE*	ATP synthase subunit c	62A>T	Asn21Ile
SP3	66	28378	missense variant	*Org1_00657*	hypothetical protein	500C>T	Thr167Ile
SP3	66	31303	missense variant	*walR*	transcriptional regulatory protein walR	319C>T	Arg107Cys
SP3	66	33408	missense variant	*pcrA*	ATP-dependent DNA helicase PcrA	761A>G	Asp254Gly
SP3	66	34705	missense variant	*ybiV*	sugar phosphatase YbiV	527C>T	Ser176Leu

Strains	Day of passage	Mutation position	Mutation type	Mutated gene	Function	Base change	Amino acid change
SP3	66	43553	missense variant	*Org1_01695*	hypothetical protein	95C>A	Pro32Gln
SP3	66	61600	missense variant	*Org1_01489*	hypothetical protein	206G>C	Gly69Ala
SP3	66	76543	missense variant	*Org1_01939*	putative universal stress protein	247G>A	Val83Ile
SP3	66	84845	missense varian	*Org1_00143*	hypothetical protein	42G>A	Met14Ile
SP3	66	127787	missense variant	*pnp_1*	polyribonucleotide nucleotidyltransferase	247C>G	Arg83Gly
SP3	100	10208	missense variant	*Org1_00874*	hypothetical protein	82G>T	Ala28Ser
SP3	100	28077	missense variant	*atpE*	ATP synthase subunit c	62A>T	Asn21Ile
SP3	100	28378	missense variant	*Org1_00657*	hypothetical protein	500C>T	Thr167Ile
SP3	100	31303	missense variant	*walR*	transcriptional regulatory protein walR	319C>T	Arg107Cys
SP3	100	33408	missense variant	*pcrA*	ATP-dependent DNA helicase *PcrA*	761A>G	Asp254Gly

Strains	Day of passage	Mutation position	Mutation type	Mutated gene	Function	Base change	Amino acid change
SP3	100	34705	missense variant	*ybiV*	sugar phosphatase YbiV	527C>T	Ser176Leu
SP3	100	43553	missense variant	*Org1_01695*	hypothetical protein	95C>A	Pro32Gln
SP3	100	52557	frameshift variant	*sasA*	Adaptive-response sensory-kinase SasA	1277delA	Asn426fs
SP3	100	76543	missense variant	*Org1_01939*	putative universal stress protein	247G>A	Val83Ile
SP3	100	84845	missense varian	*Org1_00143*	hypothetical protein	42G>A	Met14Ile
SP3	100	93138	missense variant	*tcaR*	HTH-type transcriptional regulator *TcaR*	200C>T	Ala67Val
SP3	100	127787	missense variant	*pnp_1*	polyribonucleotide nucleotidyltransferase	247C>G	Arg83Gly
SP3	100	159526	stop gained, disruptive inframe deletion	*glpK*	glycerol kinase	792_863delTGGAACTGGTGGCTTCATGTTAATGAATACAGGT-	Tyr264_Thr288delinsTer

IBG, isopropoxy benzene guanidine; SP1, SP2 and SP3 indicate three independent *S. aureus* 29213 cultures
